# Human Placental Lactogen in Relation to Maternal Metabolic Health and Fetal Outcomes: A Systematic Review and Meta-Analysis

**DOI:** 10.3390/ijms232415621

**Published:** 2022-12-09

**Authors:** Kate Rassie, Rinky Giri, Anju E. Joham, Helena Teede, Aya Mousa

**Affiliations:** 1 Monash Centre for Health Research and Implementation (MCHRI), School of Public Health and Preventive Medicine, Monash University, Level 1, 43-51 Kanooka Grove, Clayton, Melbourne, VIC 3168, Australia; 2 Department of Diabetes, Monash Health, 246 Clayton Rd, Clayton, Melbourne, VIC 3168, Australia

**Keywords:** birthweight, gestational diabetes mellitus, human chorionic somatomammotropin, human placental lactogen, type 1 diabetes mellitus

## Abstract

Human placental lactogen (hPL) is a placental hormone which appears to have key metabolic functions in pregnancy. Preclinical studies have putatively linked hPL to maternal and fetal outcomes, yet—despite human observational data spanning several decades—evidence on the role and importance of this hormone remains disparate and conflicting. We aimed to explore (via systematic review and meta-analysis) the relationship between hPL levels, maternal pre-existing and gestational metabolic conditions, and fetal growth. MEDLINE via OVID, CINAHL plus, and Embase were searched from inception through 9 May 2022. Eligible studies included women who were pregnant or up to 12 months post-partum, and reported at least one endogenous maternal serum hPL level during pregnancy in relation to pre-specified metabolic outcomes. Two independent reviewers extracted data. Meta-analysis was conducted where possible; for other outcomes narrative synthesis was performed. 35 studies met eligibility criteria. No relationship was noted between hPL and gestational diabetes status. In type 1 diabetes mellitus, hPL levels appeared lower in early pregnancy (possibly reflecting delayed placental development) and higher in late pregnancy (possibly reflecting increased placental mass). Limited data were found in other pre-existing metabolic conditions. Levels of hPL appear to be positively related to placental mass and infant birthweight in pregnancies affected by maternal diabetes. The relationship between hPL, a purported pregnancy metabolic hormone, and maternal metabolism in human pregnancy is complex and remains unclear. This antenatal biomarker may offer value, but future studies in well-defined contemporary populations are required.

## 1. Introduction

Human pregnancy is defined by complex hormonal and metabolic changes, which are essential to regulate nutrient availability and ensure the health of the mother and the growing fetus. In late gestation, a significant increase in maternal insulin resistance (prioritising the delivery of glucose and amino acids across the placenta for use by the fetus) is paralleled by a compensatory increase in insulin synthesis and secretion. The endocrine mechanisms underlying these changes are incompletely understood.

In women with obesity, pre-gestational diabetes (type 1 or 2), or polycystic ovary syndrome (PCOS); the physiological adaptations of pregnancy exacerbate the existing states of insulin resistance and/or deficiency underpinning these conditions. During pregnancy, gestational diabetes mellitus (GDM), defined as carbohydrate intolerance of variable severity with first onset or recognition during pregnancy, is also increasingly common. GDM reflects a failure to sufficiently augment insulin secretion in the face of progressive gestational insulin resistance. Maternal diabetes of any type in pregnancy increases the risk of fetal macrosomia and obstetric complications, and is associated with potential adverse long-term alterations to the metabolic profile of the mother and offspring [1]. GDM during pregnancy is a significant risk factor for future cardiovascular disease in women—one recent meta-analysis of observational trials suggested that women with a history of GDM had a twofold higher risk of future cardiovascular events compared with those who did not [2]. As such, an improved understanding of the mechanisms that alter maternal insulin resistance—and further insights into biomarkers which can facilitate early identification of women at risk of GDM—are key priorities.

Human placental lactogen (hPL), previously known as human chorionic somatomammotropin, is a polypeptide hormone produced during pregnancy by the syncytiotrophoblast cells of the placenta. A member of the somatotropin family, hPL is structurally homologous to pituitary growth hormone (GH), prolactin (PRL) and placental growth hormone (GH-V) [3]. In humans, hPL binds mainly to the PRL receptor, with lower affinity for the GH receptor [4]. Detection of hPL in maternal plasma occurs at approximately six weeks of gestation, and its concentration then increases linearly until about the thirtieth week of pregnancy, reaching peak concentrations of 5000–7000 ng/mL. The secretion rate of hPL near term is approximately 1 g/day, significantly greater than that of any other hormone [5]: indeed, the peak concentration of hPL is at least 25-fold that of PRL [6]. Maternal serum hPL levels are positively correlated with placental mass and are greater in multiple than singleton gestations [5]. hPL was widely used clinically in the 1970s–1980s, prior to widespread obstetric ultrasound, to assess fetoplacental wellbeing in late pregnancy [7,8]; but has since fallen from routine clinical use. As a pregnancy-specific hormone, hPL is rapidly washed from the maternal circulation following delivery of the placenta.

Along with estrogen, progesterone and PRL, hPL promotes third trimester mammary ductal and alveolar growth for lactogenesis (hence its designation as a ‘lactogenic’ hormone). However, hPL also has important metabolic roles in carbohydrate and lipid metabolism and fetal nutrient availability. It has been widely implicated in pregnancy-induced insulin resistance, maternal beta cell adaptation to pregnancy, and regulation of fetal growth in pre-clinical studies [3,9]. As such, altered hPL dynamics have been investigated in the context of metabolic conditions and outcomes in pregnancy.

The current literature on hPL in relation to maternal metabolism consists primarily of pre-clinical work and dated observational studies. Clinical research on hPL in human pregnancy has been relatively limited in recent decades, despite historical data suggesting that the hormone may have significant diagnostic and therapeutic potential.

In this systematic review, we examine current evidence regarding the relationship between hPL and maternal metabolic outcomes in pregnancy and postpartum, as well as key fetal outcomes, in the context of common metabolic conditions. We seek to provide mechanistic insights and examine the clinical implications of these findings.

## 2. Materials and Methods

### 2.1. Protocol and Registration

This review is part of a larger evidence synthesis examining lactogenic hormones in pregnancy and postpartum, and was conducted following the Preferred Reporting Items for Systematic Reviews and Meta-Analysis (PRISMA) 2020 guidelines. A protocol for this review is published [10] and was registered with the International Prospective Register of Systematic Reviews (PROSPERO), CRD42021262771.

### 2.2. Information Sources and Search Strategy

A systematic search strategy (Supplementary File S1) combining MeSH terms and text words was developed using the OVID platform, in consultation with expert subject librarians, and translated to other databases. MEDLINE via OVID, MEDLINE ePub ahead of print, in-process, in-data review and other non-indexed citations via OVID, CINAHL plus, and Embase were searched on 8 July 2021 (updated 9 May 2022). Bibliographies of relevant studies identified by the search strategy were also manually searched to identify additional eligible studies.

### 2.3. Eligibility Criteria

Selection criteria using a modified version of the Participant, Exposure, Comparison, Outcome and Study Type (PECOT) framework [11] were established a priori. These were used to determine the eligibility of articles.

Studies were included in the systematic review when the following criteria were fulfilled: (i) participants were pregnant women and women up to 12 months postpartum; (ii) endogenous maternal serum hPL was measured and reported at least once during pregnancy; (iii) a comparison group of any type (or no comparison group) was reported; and (iv) one of the following key outcomes was reported in relation to hPL:

Maternal:

- Diabetes status during pregnancy and up to 12 months postpartum (pre-existing diabetes [type 1 or type 2], impaired glucose tolerance, or GDM; adequately defined)
- Metabolic indices (continuous measurements) related to maternal glucose/lipid metabolism (e.g., glucose measurements on oral glucose tolerance test; insulin secretion/sensitivity/resistance indices; beta-cell function) during pregnancy or up to 12 months postpartum
- Obesity/body mass index, gestational weight gain
- Postpartum weight change
- Polycystic ovary syndrome
- Lipid profile

Infant:

- Birthweight (absolute/centiles, macrosomia), growth restriction or placental mass in relation to pregnancies affected by maternal GDM or pre-gestational diabetes.

Eligible study types included cross-sectional, longitudinal cohort or case–control studies, and randomised controlled trials. Narrative and systematic reviews were excluded from the analysis, but their bibliographies were examined to identify relevant eligible articles. Commentaries, letters, conference abstracts, and case reports were excluded. Only full text English articles were included, with no date limits for eligibility. Maternal diabetes was considered adequately defined if the study clearly referred to type 1 or type 2 diabetes mellitus (T1DM, T2DM), GDM, or impaired glucose tolerance. If definitions were less clear (for example, older studies using White’s classification of diabetes in pregnancy), studies were included only if the information provided was sufficient to confidently deduce diabetes type. If definition adequacy varied between groups, the study was included only for the group(s) meeting definition requirements.

Studies were excluded if they were animal, in vitro or tissue/cell culture studies; involved exogenous administration of hPL; involved an intervention or procedure to manipulate hPL; focused on assisted reproductive technologies; or focused primarily on women with other pregnancy pathologies (e.g., pre-eclampsia, fetal death).

### 2.4. Study Selection and Risk of Bias Assessment

**Figure 1:**
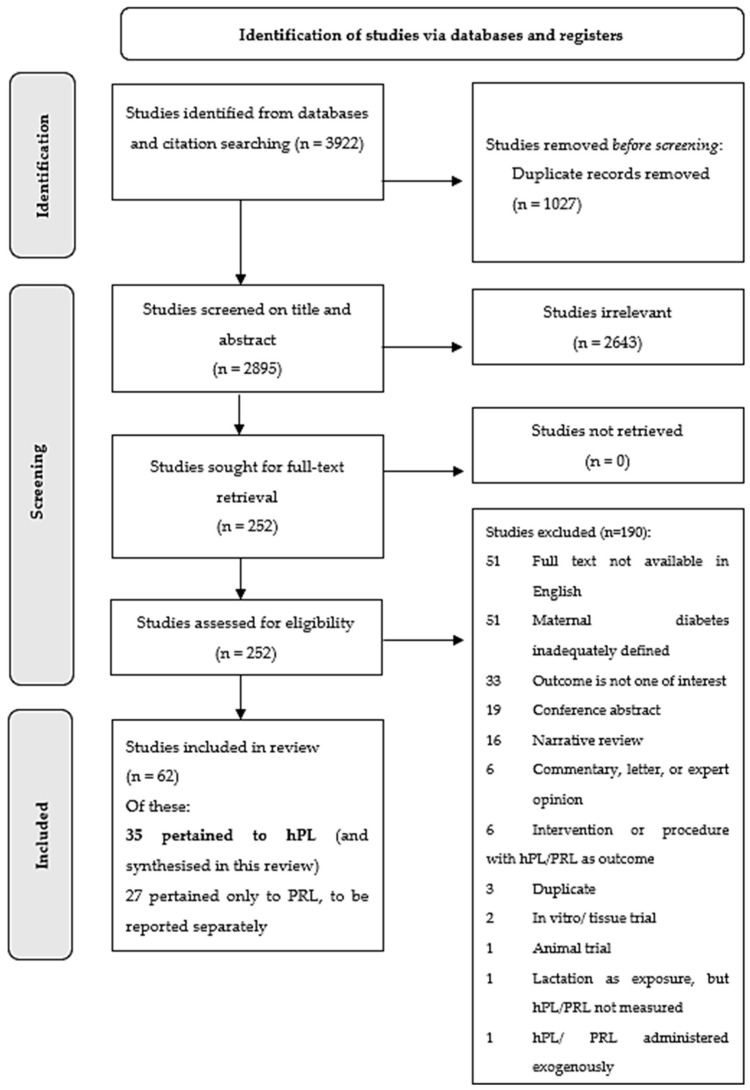
PRISMA flowchart.

Two independent reviewers (KR and RG) screened all abstracts and full texts (Figure 1) and performed quality assessment, with 10% of studies assessed in duplicate. Quality appraisal (risk of bias) was conducted on Covidence software, using the Monash Centre for Health Research and Implementation (MCHRI) Evidence Synthesis Program tool [12], based on the Newcastle-Ottawa Scale for non-randomised studies [13]. Quality items were assessed using a descriptive component approach to evaluate external validity (study design, inclusion/exclusion criteria, and appropriateness of measured outcomes) and internal validity (selection, performance, detection, and reporting biases; attrition, confounding, statistical methodology, and study power). Studies that fulfilled all, most or few criteria were deemed to have low, moderate, or high risk of bias, respectively. Discrepancies were resolved through discussion and consensus.

### 2.5. Data Extraction and Synthesis

Data were manually extracted from all included studies by two independent reviewers using a purpose-built data extraction form in Microsoft Excel, with 10% extracted in duplicate. Information was collected on general study characteristics (authors, publication year and source, country, study design, duration of follow-up), participant characteristics (baseline age, metabolic conditions, parity, body mass index (BMI), ethnicity), hPL timepoints and values, hPL assay techniques, key maternal outcomes assessed in relation to hPL (unadjusted and adjusted, with consideration of covariates used), key relevant infant outcomes, and conclusions.

### 2.6. Evidence Synthesis and Statistical Analysis

**Figure A1:**
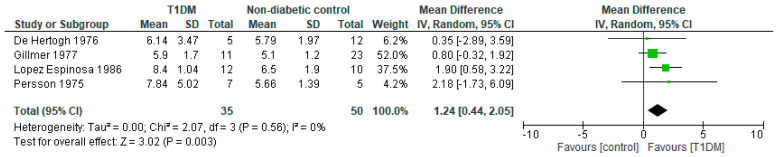
Meta-analysis of hPL levels in T1DM vs. non-diabetic control women in late pregnancy (>24 weeks)—4 studies.

**Figure A2:**
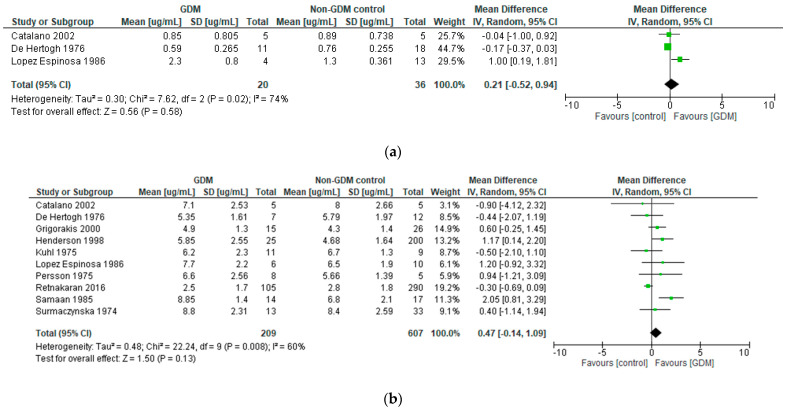
(**a**): Meta-analysis of hPL levels in GDM vs. non-diabetic control women in early pregnancy (≤24 weeks)—3 studies. (**b**): Meta-analysis of hPL levels in GDM vs. non-diabetic control women in late pregnancy (>24 weeks)—10 studies.

Where meta-analysis was possible, Review Manager 5.4.1 software was used. Where published papers contained insufficient data to be entered into meta-analysis, details were sought from the authors. Weighted mean differences (WMD) were generated using random effects models. Statistical heterogeneity was assessed using the I^2^ test, with I^2^ values of >50% indicating moderate to high heterogeneity. Sensitivity analyses were conducted to examine the effects of studies with high risk of bias on the overall results. Sensitivity analysis was also performed with exclusion of studies published prior to the year 2000 (given that older studies likely reflect a different clinical and therapeutic environment). Where meta-analysis was not possible, narrative synthesis of results was performed. Data are presented in summary tables and in narrative format. Forest plots (Figure A1 and Figure A2a,b) and funnel plots (Supplementary File S2) were used to present the results of meta-analyses and publication bias assessments, respectively.

## 3. Results

### 3.1. Study Selection and Characteristics

A total of 3922 results were retrieved from the initial database search for the broader evidence synthesis examining lactogenic hormones. Following removal of duplicates, 2643 and 190 studies were excluded at abstract and full text screening, respectively (Figure 1). Of note, studies excluded on the basis of unavailable English full text (*n* = 51) or inadequate maternal diabetes definition (*n* = 51) were disproportionately dated, with all published prior to 1998 and 2000, respectively.

A total of 62 studies met the broader eligibility criteria for inclusion, of which 35 pertained specifically to hPL and were included in the current review. Due to methodological heterogeneity, meta-analysis was only possible for hPL differences in late pregnancy by T1DM status (four studies) and for hPL differences in early and late pregnancy by GDM status (three and 10 studies, respectively).

### 3.2. Risk of Bias of Included Studies

Of the 35 included studies, 12 were deemed high risk of bias, 16 moderate, and seven low (Table A1, Table A2, Table A3, Table A4 and Table A5). The main aspects contributing to high risk of bias were the presence of confounding and selection bias, both of which were present in seven of the 12 studies deemed high risk of bias. Low-quality statistical analysis and concerns regarding the accuracy and validity of hormone measurement were also common domains of concern, each present in six of the 12 high-risk studies.

Based on visual inspection of funnel plots, there was no evidence of publication bias across any of the outcomes assessed (Supplementary File S2).

### 3.3. Synthesis of Results

#### 3.3.1. Human Placental Lactogen in Pregnancies Affected by Pre-Gestational Metabolic Conditions

Twelve studies examined hPL across pregnancy in women with adequately-defined pre-gestational diabetes mellitus (PGDM) (Table A1). These were all published prior to 1998 and focused on T1DM, with no studies in pregnancies affected by T2DM or PCOS.

(a) Differences in hPL between T1DM and control pregnancies

Seven of the 12 studies [14,15,16,17,18,19,20] reported measuring early pregnancy (≤24 weeks) hPL in women with T1DM compared with controls, although only six clearly reported between-group hPL results. Of these, four found hPL to be significantly lower in T1DM than controls for at least one early pregnancy timepoint [14,15,16,17]. One found hPL to be higher in T1DM than controls [18], while the other found no difference [20]. With the exception of one study [16], all of these studies lacked sufficient raw published data for meta-analysis, and their age precluded contacting authors for more details.

Nine studies compared late pregnancy (>24 weeks) hPL between T1DM and controls. Of these, four [16,18,21,22] had available data for meta-analysis (Figure A1). Latest available pregnancy measurements were used in all cases (all 34–40 weeks). Pooled results showed significantly higher late pregnancy hPL levels in women with T1DM than controls (WMD = 1.24 µg/mL, 95% CI 0.44 to 2.05, *p* = 0.003), with no heterogeneity (I^2^ = 0%, *p* = 0.6). Sensitivity analysis with exclusion of the two studies deemed high risk of bias did not significantly alter results. Of the five studies without sufficient information for inclusion in the meta-analysis; two showed significantly higher late hPL levels in T1DM than control pregnancies [19,23], two showed no difference [15,20], and a single small study showed lower late hPL in T1DM than controls [14].

Two studies which sub-divided T1DM subjects by class (White’s classification, based on complications and duration) found no difference in hPL values across subgroups of progressive T1DM ‘severity’ [17,18]. However, a third study (focused specifically on diabetic retinopathy) found higher hPL values in the subset of pregnant women with T1DM with retinopathy, particularly progressive retinopathy, who also had worse glycaemic control and higher placental mass [19].

(b) Relationship between hPL and glycaemic measures in T1DM

Five studies [14,15,18,21,22] examined hPL in T1DM in relation to plasma glucose (mean and/or prevailing). Botta et al. [14] reported that hPL was inversely associated with blood glucose levels across gestation (both average glucose that day and glucose at the time of hPL sampling). The remaining studies, of which two had similar methodology [15,18] and two sampled hPL serially over 8–24 h [21,22] reported no relationship between hPL and glucose.

Two studies examined HbA1c in relation to hPL in T1DM (one in a one-off early pregnancy sample, one at serial timepoints); both showing no significant relationship [15,17].

Three studies examined hPL levels relative to the increase in insulin requirements across pregnancy in T1DM, all reporting no relationship between these two variables [18,20,24]. In contrast, the sole study to use insulin clamp methodology to directly quantify insulin resistance in early and late pregnancy found that the size of hPL increment across pregnancy was significantly inversely proportional to late pregnancy insulin sensitivity in women with T1DM (*n* = 6) [25].

#### 3.3.2. Human Placental Lactogen in Pregnancies Affected by Gestational Diabetes Mellitus

Seventeen studies examined hPL across pregnancy in women with GDM (Table A2).

(a) Differences in hPL between GDM and control pregnancies

Five studies [16,18,26,27,28] compared hPL in women with GDM and controls in early pregnancy (≤24 weeks, often prior to GDM diagnosis and recognition). In the three studies with sufficient data for meta-analysis (Figure A2a), pooled analysis showed no significant difference in early pregnancy hPL between GDM and control pregnancies (WMD = 0.21 µg/mL, 95% CI −0.52 to 0.94, *p* = 0.6). Statistical heterogeneity was high (I^2^ = 74%, *p* = 0.02), and small sample sizes universal. Two of the three studies [16,26] were deemed high risk of bias; precluding sensitivity analysis. Of the two studies with insufficient detail for inclusion in the meta-analysis, one reported higher hPL values in women with GDM than controls [28], and the other showed no significant difference herein [27].

Fourteen studies compared hPL between women with GDM and controls in later pregnancy (>24 weeks), twelve of which were eligible for meta-analysis, but two were subsequently excluded due to significant methodological concerns and a suspicion of erroneous hPL values (see Table A2 footnotes) [29,30]. All values were third trimester samples. Pooled analysis of the ten included studies [16,18,22,26,31,32,33,34,35,36] using the latest timepoint if multiple were available (Figure A2b), suggested no significant difference in late pregnancy hPL between women with GDM and controls (WMD = 0.47 µg/mL, 95% CI −0.14 to 1.09, *p* = 0.1), with moderate heterogeneity (I^2^ = 60%, *p* = 0.008). Sensitivity analyses with exclusion of older studies and those deemed high risk of bias did not significantly alter results. The two studies that lacked sufficient detail for inclusion in the meta-analysis also found no significant difference in late hPL between GDM and controls [37,38].

Three studies examined the clinical utility of hPL as a risk predictor for GDM. Two studies (one with major methodological limitations, see Table A2 footnotes [29]) suggested it was unlikely to be useful, due to poor classification performance [28] or non-significant predictive capacity [29]. Conversely, the third study [34] suggested that hPL may be a promising adjunct to screening glucose challenge tests in predicting the likelihood of a subsequent abnormal oral glucose tolerance test (OGTT).

(b) Relationship between hPL and glycaemic measures in GDM

Nine studies examined hPL in relation to cross-sectional or longitudinal glycaemic parameters in GDM such as plasma glucose or insulin, or markers of insulin sensitivity or resistance. Overall, none found consistent relationships between hPL and these variables in women with GDM or controls [18,22,29,30,32,33,36,38,39].

#### 3.3.3. Human Placental Lactogen in Relation to Glycaemic or Insulin-Related Parameters in Healthy Pregnancies and Postpartum

Four studies [40,41,42,43] examined hPL in relation to glycaemic or insulin-related parameters in healthy pregnant women (Table A3). The methodology of these studies varied considerably, precluding meta-analysis. Benny et al. [40] examined hPL dynamics across a 24 h period in the third trimester, showing a peak after overnight fasting (temporally coincident with the time of lowest glucose and insulin, and potentially consistent with the idea of hPL as an insulin-antagonistic hormone). Enzi et al. [41] found that maternal hPL levels at 34 weeks were positively related to the area under the curve (AUC) of both glucose and insulin, suggesting this confirmed the diabetogenic effects of hPL. This differed from the findings of two other studies, where hPL was unrelated to prevailing glucose [42] or to 2 h OGTT insulin or glucose levels [43] in healthy pregnancies. However, higher hPL levels were associated with higher levels of non-esterified fatty acids in one study [42], suggestive of anti-insulin, diabetogenic properties.

One study [44] related hPL to postpartum glycaemia, finding that hPL in late pregnancy was not an independent predictor of insulin resistance, beta-cell function or diabetes risk (all measured at 3 months postpartum).

#### 3.3.4. Human Placental Lactogen in Relation to Body Mass Index or Gestational Weight Gain in Pregnancy

Four studies [30,41,45,46] examined hPL in relation to maternal BMI or gestational weight gain (GWG) (Table A4). Two studies showed no relationship between hPL and maternal BMI [30] or hPL and GWG (crudely categorised as <20% or >20% ideal body weight for normal or excessive GWG, respectively) [41]. Lin et al. [45] described an inverse relationship between hPL and absolute maternal weight at term, proposed to be a dilutional effect (more tissue space in larger women). McCarrick et al. [46] found that obese women were over-represented in a group of women with low hPL but normal estrogen levels, suggesting that obesity may impact on hPL regulation and activity (although many of these women had other pregnancy complications which may have explained their low hPL levels, such as toxaemia or intra-uterine growth restriction).

#### 3.3.5. Human Placental Lactogen in Relation to Fetal, Neonatal or Placental Outcomes in Pregnancies Affected by Maternal Diabetes

Seven studies [14,18,20,35,38,47,48] examined hPL in relation to fetal, neonatal or placental outcomes in pregnancies affected by maternal PGDM/GDM (Table A5). Variable methodology and lack of reporting detail prevented meta-analysis.

One study [48] examined hPL in relation to fetal growth/size in the early stages of T1DM pregnancies (*n* = 26), and found that hPL at 7–16 weeks could be best related to menstrual age when the latter was corrected by any ultrasonographically determined ‘growth delay’. Given that hPL reflects functional trophoblastic mass, this suggested that the observed growth delay in T1DM pregnancies may relate to delayed placental development.

Three studies examined hPL in the late third trimester relative to placental weight at delivery in pregnancy cohorts affected by (adequately-defined) maternal PGDM/GDM. One study [18] of 38 women found that late pregnancy hPL was strongly positively correlated to placental weight in T1DM (r = 0.8, *p* < 0.01), GDM (r = 0.6, *p* < 0.05), and controls (r = 0.6, *p* < 0.05). In the remaining two studies, one found no relationship (despite a trend noted) between late pregnancy hPL and placental weight in a small combined cohort of 15 T1DM and 10 control pregnancies [14] and the other found that hPL was positively associated with placental mass in the larger control group (*n* = 69; r = 0.3, *p* < 0.01) but not in the T1DM cohort (*n* = 40), likely due to low statistical power [20].

Five studies examined hPL in relation to infant birthweight in pregnancies affected by PGDM/GDM. Two [14,35] found no relationship between hPL at 36 weeks or at term with birthweight in a combined cohort of women with T1DM and controls (*n* = 25) or a combined cohort of women with GDM, women with premature deliveries and controls (*n* = 46), respectively. Conversely, two other studies showed positive relationships between third trimester hPL and corrected birthweight in T1DM (r = 0.48, *p* < 0.02) [20] or birthweight in a GDM cohort (r = 0.59, *p* < 0.05) [38]. Finally, Small et al. [47] examined hPL in relation to birthweight ‘class’, finding that a T1DM group with macrosomic infants (mean birthweight 3.96 kg at 37 weeks) had significantly higher hPL at 34 weeks than matched T1DM pregnancies without macrosomia (mean birthweight 3.05 kg at 37 weeks).

## 4. Discussion

To our knowledge, this is the first systematic review of hPL in relation to maternal metabolic outcomes in pregnancy. Specifically, we explored hPL in healthy pregnancies and in those with PGDM/GDM, and relationships to maternal metabolic parameters and fetal growth within these subgroups. Systematic review and meta-analysis suggests altered hPL dynamics in pregnancies affected by T1DM, but no relationships with GDM were identified. hPL appears positively correlated with placental mass in PGDM/GDM and elevated in pregnancies affected by macrosomia. However, hPL levels were not clearly linked to maternal glycaemic outcomes in PGDM/GDM, despite pre-clinical evidence for physiological roles in both insulin resistance and maternal beta-cell adaptation to pregnancy.

### 4.1. hPL in Pre-Gestational (Type 1) Diabetes Mellitus

In pre-gestational T1DM, the results of our review suggest altered hPL dynamics across gestation (with differential effects in early and late pregnancy). Meta-analysis comparing early hPL levels in T1DM vs. control pregnancies was not possible due to a lack of detailed comparative data, but results were broadly suggestive of lower early hPL levels in pregnancies affected by T1DM. Whilst some authors have speculated that these low levels may be a direct response to maternal hyperglycaemia [14], it should be noted that experimental evidence showing depression of hPL levels required maternal blood glucose to be raised dramatically (via rapid intravenous infusion over 30 min to a mean of 22.2 mmol/L, in the seminal trial) [49]. The evidence summarised in our review suggests that more subtle alterations of plasma glucose, such as may occur in adequately-controlled maternal T1DM, are unlikely to have a major direct impact on hPL levels. Overall, the lower levels of hPL observed in early T1DM pregnancy seem more likely to relate to delayed trophoblastic development [16,48]. Such a mechanism would be consistent with the observation that concentrations of other key gestational hormones–such as PRL and human chorionic gonadotropin (hCG)–may also lag behind normal early pregnancy reference ranges in T1DM pregnancies, particularly in the context of suboptimal glycaemic control [50].

In the later part of T1DM pregnancy (namely the third trimester), the results of our review and meta-analysis support higher circulating maternal hPL levels in T1DM than control pregnancies. The observation of higher hPL levels in T1DM has typically been attributed to greater placental mass in such pregnancies. Mechanistically, this may reflect fetal hyperglycaemia with secondary hyperinsulinaemia, leading to macrosomic stimulation of the placenta [8]. Early clinical literature, dating to the era where the hormone was in routine obstetric use, is in keeping with this: high-normal or high hPL levels were “expected” in T1DM pregnancy. When levels fell below this, they were likely to be suggestive of a separate superimposed reason for fetoplacental compromise (such as toxaemia or late fetal demise, which were common occurrences in T1DM cohorts in that era) [51].

Together, the results of studies of hPL in T1DM suggest that the relationship between hPL and maternal metabolism is likely bidirectional. Whilst hPL certainly has metabolic actions, its concentrations are also likely influenced by an altered maternal metabolic environment (such as in T1DM), with different mechanisms operational in early vs. late pregnancy.

### 4.2. hPL in Maternal Glycaemia and Gestational Diabetes Mellitus

Our meta-analysis of studies comparing absolute hPL concentrations between women with GDM and controls in both early and late pregnancy showed no statistically significant differences between groups. Similarly, the small number of studies which investigated hPL as a GDM risk prediction biomarker do not support its predictive utility.

A body of pre-clinical evidence certainly provides theoretical grounds to suggest that hPL may be immediately relevant to maternal glucoregulation in pregnancy: at high concentrations, hPL has classically been considered a ‘diabetogenic’ hormone [8,52] with insulin-antagonistic and lipolytic effects. Most endocrine texts still describe hPL as a key contributor to gestational insulin resistance, increasing fetal nutrient availability by sparing glucose, amino acids and ketones for placental-fetal transport [53]. However, rodent and in vitro human data have also repeatedly identified a key parallel role for hPL (acting via the PRL receptor) to induce maternal pancreatic adaptation to pregnancy, increase beta-cell mass, and potentiate glucose-stimulated insulin secretion [6,54,55].

Despite these roles, the human data synthesised in our review suggests that absolute maternal hPL concentrations measured in human pregnancy populations may be difficult to link directly to glycaemic parameters. As such, it seems likely that the in vivo metabolic effects of hPL are likely to be much more complex than suggested by existing pre-clinical evidence, much of which was accumulated a generation ago. For example, there is increasing acknowledgment of the multifactorial and synergistic nature of late pregnancy insulin resistance, with important roles for GH-V (a powerful lipolytic hormone), maternal insulin like growth factor 1 (IGF-1), progesterone, cortisol and tumor necrosis factor (TNFα); as well as a fall in adiponectin [9]. As such, the designation of hPL as the “major diabetogenic stress factor of pregnancy” may be overly simplistic. Similarly, autopsy evidence from the pancreata of pregnant women indicates that the adaptive beta cell changes of human pregnancy may be less profound and different in nature to those observed in rodents [56], which immediately suggests that the extrapolation of findings from sub-primate models about the insulinogenic properties of the hormone must be approached with caution. Furthermore, circulating serum levels of a hormone do not always tell the whole story: for example, recent work has suggested that certain PRL receptor polymorphisms may predict GDM risk, implying that differences in hormone action—at a tissue level—may be just as important as absolute hormone concentrations [57].

Thus, whilst pre-clinical studies clearly support a key role for hPL in metabolic adaptations to human pregnancy (both as an insulin antagonist and as a stimulus for augmented insulin secretion); the collated observational data suggest that its measured circulating concentrations may not provide direct insights into maternal glucose homeostasis.

### 4.3. hPL in Fetal Growth in Pregnancies Affected by Maternal Diabetes

Studies have consistently demonstrated a positive association between hPL and placental mass (in both diabetic and non-diabetic cohorts) [14,18,51,58,59] and a positive association between hPL and infant birthweight has also been demonstrated in several large general pregnancy cohorts [60,61,62]. Our review, which was limited to pregnancies affected by adequately-defined maternal diabetes, generally supported these findings. Accurate antenatal prediction of fetal macrosomia remains challenging, and current strategies (including fundal measurements and ultrasound assessment) are resource-intensive. There is thus a clear requirement for maternal serum biomarkers in improving macrosomia prediction, particularly in women at high risk (such as those with PGDM/GDM). Whilst several biomarkers have been assessed for their association with birthweight or macrosomia (both in diabetic and non-diabetic pregnancies), evidence is mixed and uncertainties around clinical utility persist [63]. hPL has recently been largely overlooked in this capacity, but previous work suggests it may have significant potential if revisited [19,47].

Mechanistically, a direct role for hPL in the regulation of fetal growth is also feasible: for example, targeted reductions in placental lactogens in sheep pregnancy via modification of placental gene expression result in significantly reduced fetal weight, possibly mediated by disrupted IGF-1 and IGF-2 expression [64,65]. In humans, low levels of hPL in small for gestational age pregnancies are commonly observed (along with reduced levels of GH-V) [62,66,67]. The role of hPL in large for gestational age (LGA) pregnancies—particularly those affected by maternal metabolic disease—is similarly interesting. In general obstetric populations, significant positive relationships between maternal hPL levels at 34 weeks’ gestation and neonatal body weight, body fat mass and fat cell weight have been reported [41], and other research has demonstrated a 1.6-fold higher expression of hPL genes in the placentas of LGA newborns compared to those of normal size [67]. As such, hPL may contribute aetiologically to macrosomia, aside from simply reflecting increased placental mass in LGA pregnancies. Whilst fetal overgrowth in maternal obesity and diabetes is commonly associated with placentomegaly, it is also possible that the resulting hPL excess may further stimulate both maternal and fetal beta-cell expansion and increase fetal insulin production, which would promote glycogenesis, fat deposition and fetal growth [9].

Given the likely positive relationships between hPL and placental mass/neonatal weight in pregnancies affected by maternal diabetes, as well as a possible aetiological role in the development of macrosomia; late-pregnancy hPL warrants re-visiting in modern obstetric populations (both with and without diabetes).

### 4.4. Strengths and Limitations

As noted above, this is the first review to systematically collate and synthesise the literature linking hPL to maternal metabolic outcomes in pregnancy and related fetal outcomes. We employed rigorous, international gold-standard methodology with a protocol developed a priori to ensure transparency. The review addresses a broad, mechanistic question that links important aspects of female reproductive and metabolic health; and sheds light on a hormone which has been overlooked in the endocrine literature in recent decades. Identification of biomarkers that may aid with GDM risk prediction, or help with identifying complications in pregnancies affected by maternal diabetes; is a key health priority–particularly given the accumulating body of evidence linking gestational metabolic disease (and insulin resistance) to lifetime metabolic and cardiovascular risk in women.

Limitations of the review process include restriction of the search to published English language articles. In addition, the requirement for clearly defined maternal diabetes type excluded some older studies (pre-1980s, often referring only to ‘maternal diabetes’). Inclusion of this literature would have increased the number of included studies–and possibly numbers for meta-analysis–but would have introduced significant uncertainty and made results less applicable to modern clinical populations.

Limitations of the literature were substantial and precluded firm conclusions regarding the role of hPL in pregnancy and postpartum in the context of common metabolic conditions. These limitations included heterogeneous methodology and a frequent lack of detail in data reporting, which contributed to the inability to perform meta-analysis for many outcomes. Variable study quality was reflected in the risk of bias assessments (28 of 35 studies were deemed to have moderate or high risk of bias). Studies were small and were all observational in nature, increasing the likelihood of low statistical power and residual confounding. Measurement of hPL at only one or two timepoints (and often within a broad gestational age bracket, without subsequent correction for exact gestational age) was a significant limitation of many studies, given the steep increase in hPL concentrations known to occur across normal pregnancy. BMI is also an important potential confounder in the relationship between hPL and metabolic indices, but BMI reporting in the included studies was variable, and precluded stratification of meta-analyses based on BMI. Data (on T1DM, in particular) was dated; and thus reflected a historical therapeutic environment. Assay methodology for measuring hPL also varied, with older studies using radioimmunoassay techniques and newer studies favouring enzyme-linked immunoassays. There were no data relating hPL to maternal metabolic outcomes or fetal parameters in T2DM or PCOS cohorts, or to lipid profiles; and data on maternal obesity and GWG was sparse. Finally, the hormonal environment of pregnancy and postpartum is complex, and studies focusing on absolute levels of a single hormone may overlook other factors such as hormone synergy, local tissue levels, and receptor polymorphisms.

## 5. Conclusions

In summary, the findings of our review suggest that in T1DM pregnancies, hPL levels may be lower than controls in early pregnancy (possibly reflecting delayed placental development) and higher than controls in later pregnancy (likely in keeping with higher placental masses), but that absolute hPL concentrations are not clearly linked to maternal glycaemic outcomes in PGDM or GDM, nor to GDM status/risk. Moreover, hPL is likely positively related to placental mass and infant birthweight in pregnancies affected by PGDM or GDM, and may be aetiologically important in the regulation of fetal growth. Despite having fallen from routine clinical use in recent decades, hPL may warrant renewed investigation as an antenatal biomarker for the prediction of macrosomia. However, given the limited available data, small study numbers, and substantial heterogeneity in study design and methodology, future high-quality studies exploring this hormone in well-defined contemporary populations are required to clarify these relationships and to inform future research and clinical practice.

## Supplementary Materials

The following supporting information can be downloaded at: https://www.mdpi.com/article/10.3390/ijms232415621/s1.

## Appendix A

**Table A1:** Studies examining hPL in pregnancies affected by pre-gestational type 1 diabetes mellitus—12 studies.

Author and Year; Country of Origin	Design	Participants and Sample Size	Methodology and hPL Pregnancy Timepoints	Metabolic Parameters Analysed	Results	Authors’ Conclusions	Risk of Bias Rating
Botta et al., 1984 [14] Italy	Longitudinal observational	*n* = 15 T1DM *n* = 10 controls	hPL sampled at: 12 weeks 16 weeks 20 weeks 24 weeks 28 weeks 32 weeks 36 weeks	T1DM status Blood glucose	Mean hPL sig lower in T1DM women than controls at 6 of the 7 timepoints (all other than 16 weeks). In T1DM women, at 5 of the 7 timepoints, sig inverse relationship between hPL and mean blood glucose that day. In T1DM women, at 4 of the 7 timepoints, sig inverse relationship between hPL and blood glucose at the time.	hPL lower in T1DM subjects than controls across preg Indication of inverse relationship between hPL and prevailing blood glucose levels in T1DM, suggesting that hPL may be influenced by hyperglycaemia, and that normalising control may normalise hPL.	Moderate
Braunstein et al., 1989 [15] USA	Longitudinal observational	*n* = 35 T1DM *n* = 31 controls	hPL sampled at: 5–6 weeks 7–8 weeks 9–10 weeks 12–13 weeks 20 weeks 27–29 weeks 35–37 weeks	T1DM status Blood glucose HbA1c	Mean hPL sig lower in T1DM women than controls at 9–10 weeks and 20 weeks (2 of 7 timepoints), at other timepoints no sig difference. Mean fasting glucose, mean 1 h post-prandial glucose and HbA1c at each gestation not related to hPL in T1DM women.	Finding of lower hPL in T1DM women at 2 timepoints likely due to chance–overall, ‘no consistent finding’ of differences between T1DM and control women. No relationship between glycaemia and hPL in T1DM at any timepoint.	Moderate
De Hertogh et al., 1976 [16] Belgium	Longitudinal observational	*n* = 21 T1DM *n* = 22 controls	hPL sampled at: 5–8 weeks 9–12 weeks 13–16 weeks 17–20 weeks 21–24 weeks 25–28 weeks 29–32 weeks 33–36 weeks 37–40 weeks	T1DM status	Mean hPL sig lower in T1DM women than controls at 13–16, 17–20 and 21–24 weeks. No sig differences at other timepoints, although three individual T1DM women had very high (outlying) hPL values in late preg.	hPL lower in T1DM subjects than controls in early preg, possibly reflecting delayed placental development. No significant differences later in preg, although levels very high in some T1DM individuals.	High
Gillmer et al., 1977 [21] UK	Cross-sectional	*n* = 11 T1DM *n* = 23 controls	hPL sampled every 1–2 h over one 24 h period in at 34–35 weeks	T1DM status Blood glucose OGTT glucose	Mean hPL over 24 h was higher in T1DM than controls, but did not reach sig (T1DM vs. controls 5.9 ± 1.7 vs. 5.1 ± 1.2 µg/mL, *p*-value NR). No sig correlation between mean hPL over 24 h and mean glucose over 24 h in any group (T1DM or controls). In control group, no sig alteration in hPL over course of OGTT (T1DM women did not have OGTT).	NS trend to higher hPL in third trimester in T1DM women than controls. hPL varied across day in all women, but no consistent relationship to meals/fasting, etc. No sig relationship between mean glucose and hPL in either group, and no hPL alteration with OGTT in controls.	Moderate
Larinkari et al., 1982 [19] Finland	Longitudinal observational	*n* = 57 T1DM (*n* = 42 with no DR, 7 with NPDR, 8 with PDR) *n* = 58 early preg controls *n* = 24 later preg controls	hPL sampled at: 7–13 weeks 14–19 weeks 20–25 weeks 26–31 weeks 32–37 weeks	T1DM status T1DM control	Mean hPL sig higher in T1DM than controls at 34–36 weeks (10.4 vs. 7.18 µg/mL, *p* < 0.001). Other time frames NR. T1DM patients with DR (*n* = 15) had higher hPL than T1DM patients without DR (*n* = 42) at both 14–19 weeks and 20–25 weeks (NS diff at other timepoints). This group had had worse glycaemic control in the first trimester. T1DM patients with DR in whom retinopathy progressed during preg (8 of 15, all with worse control and larger placental masses) all had hPL values at or above ULN after 28 weeks.	Higher hPL in late preg in T1DM subjects than controls. Within T1DM, patients with poor control and DR had higher hPL in second trimester than those without DR; and the subset with progressive DR had markedly elevated hPL values in the third trimester (in association with poor control and placentomegaly).	High
Lopez-Espinosa et al., 1986 [18] Scotland	Longitudinal observational	*n* = 15 T1DM *n* = 14 controls	For T1DM, fortnightly hPL samples 12–32 weeks and then weekly until delivery For controls, hPL monthly	T1DM status T1DM severity/complications T1DM duration Blood glucose Insulin requirements	Mean hPL in T1DM sig higher than controls in 2nd trimester (mean ± SEM 1.7 ± 0.1 vs. 1.3 ± 0.1 µg/mL, *p* < 0.01), NS diff in early 3rd trimester (6.4 ± 0.4 vs. 5.4 ± 0.5, NS), and sig higher again at 37–40 weeks (8.4 ± 0.3 vs. 6.5 ± 0.3, *p* < 0.01). No differences in hPL between T1DM subjects with differing T1DM severity/complications (White’s classes B, C and D). No relationship of hPL to duration of T1DM. No relationship of hPL to either plasma glucose levels or insulin requirements across preg in T1DM.	hPL appears higher in T1DM than control patients across late preg. No apparent relationship between hPL and T1DM plasma glucose, insulin requirements, disease duration or severity.	Moderate
Madsen et al., 1983 [23] Denmark	Cross-sectional	*n* = 42 T1DM *n* = 20 controls	One-off hPL sample at 30–36 weeks	T1DM status	Median hPL higher in T1DM than controls in 3rd trimester, 6.9 vs. 6 µg/mL; unclear if sig (*p*-value NR).	Median hPL value higher in T1DM than control women in third trimester, but unclear if sig.	High
Pedersen et al., 1998 [17] Denmark	Cross-sectional	*n* = 79 T1DM *n* = 93 controls	One-off hPL sample at 8–13 weeks	T1DM status T1DM severity/complications HbA1c	hPL value, as MoM for exact gestation, sig lower in T1DM than controls at 8–13 weeks: median difference 0.34, *p* < 0.00001 No differences in hPL between T1DM subjects with differing T1DM severity/complications (White’s classes B, C and D). No relationship of hPL to HbA1c in T1DM subjects.	hPL sig lower (for gestation) in T1DM than control preg in first trimester. Authors suggest that this may reflect delayed placental development/depressed trophoblast function in T1DM preg; and/or effect of hyperglycaemia on hPL secretion. No apparent relationship between hPL and T1DM severity/class, nor HbA1c.	Moderate
Persson et al., 1975 [22] Sweden	Cross-sectional	*n* = 7 T1DM *n* = 5 controls	Five samples of hPL over one 8 h period at 34–37 weeks	T1DM status Blood glucose FFAs/glycerol Ketones Insulin	Mean hPL over 8 h sampling period was not sig different between T1DM vs. controls. More variability in hPL noted in T1DM. hPL changes over the sampling period bore no relationship to changes in glucose, FFAs, ketones, or insulin over sampling period.	hPL in over 8 h in third trimester not sig different between T1DM and controls; although possibly more variable in T1DM. hPL not clearly related to insulin or glucose dynamics over an 8 h period in T1DM or controls.	High
Schmitz et al., 1985 [25] Denmark	Longitudinal observational	*n* = 6 T1DM	hPL sampled in early preg (~13 weeks) and late preg (~34 weeks)	T1DM insulin sensitivity (glucose disposal via clamp)	Increase in hPL from early to late preg, ΔhPL, calculated. ΔhPL inversely proportional to the degree of insulin sensitivity by late preg, ie those with larger ΔhPL were less insulin sensitive in late preg; r = −0.84, *p* < 0.04.	hPL increase inversely proportional to late preg insulin sensitivity in small T1DM clamp study cohort. Authors conclude that hPL seems to be a major factor causing impaired insulin action in late gestation.	Moderate
Spellacy et al., 1973 [24] USA	Longitudinal observational	*n* = 22 T1DM	Frequent hPL sampling from early first trimester to delivery	T1DM insulin requirements	Insulin requirement increase across pregnancy in T1DM was individually variable and not consistently related to hPL increase.	hPL rise across pregnancy showed no consistent relationship to rising insulin requirements in T1DM. Authors suggest that hPL may not be the key diabetogenic stress factor of pregnancy as previously postulated, rather increasing insulin resistance with gestation likely to be multifactorial/synergistic.	High
Stewart et al., 1989 [20] UK	Longitudinal observational	*n* = 40 T1DM *n* = 69 controls	Frequent hPL sampling between 6 and 38 weeks in T1DM subjects	T1DM status T1DM insulin requirements	hPL levels in T1DM subjects not sig different from those of controls across preg, including when T1DM sub-grouped according to study site and/or glycaemic control. Insulin requirement increase across pregnancy in T1DM not consistently related to hPL.	hPL NS different between T1DM and controls across gestation, and not related to increase in insulin requirements across gestation in T1DM. Authors state that hormonal response of T1DM women to pregnancy is not different to that of normal controls to any marked extent, and other factors likely explain their increasing insulin requirements and tendency to macrosomia.	Low

Abbreviations: hPL = human placental lactogen, NR = not reported, NS = not significant, sig = significant, SEM = standard error of the mean, MoM = multiples of the median, DR = diabetic retinopathy, NPDR = non-proliferative diabetic retinopathy, PDR = proliferative diabetic retinopathy, FFAs = free fatty acids, T1DM = type 1 diabetes mellitus, OGTT = oral glucose tolerance test, ULN = upper limit of normal, USA = United States of America, UK = United Kingdom. Data are presented as mean ± SD unless otherwise specified in the table.

**Table A2:** Studies examining hPL in pregnancies affected by gestational diabetes mellitus—17 studies.

Author and Year; Country of Origin	Design	Participants and Sample Size	Methodology and hPL Pregnancy Timepoints	Metabolic Parameters Analysed	GDM Definition Used	Results	Authors’ Conclusions	Risk of Bias Rating
Al Busaidi et al., 2004 [27] Oman	Longitudinal observational	*n* = 200, of which *n* = 15 developed GDM	One-off hPL sample at 11–13 weeks	GDM status	NR	Mean 11–13 week hPL in women who developed GDM (*n* = 15) NS diff to that of women with other preg complications (eg. PIH, IUGR) and/or control women with no complications	Early (11–13 week) hPL NS different between GDM women and controls. Early hPL does not appear to be a useful biomarker for prediction of GDM risk.	High
Al-Hussein et al., 2021 [30] † Iraq	Cross-sectional	*n* = 40 GDM (20 male fetus, 20 female) *n* = 40 controls (20 male fetus, 20 female)	One-off hPL sample, exact timepoint NR (presume >24–28 weeks after OGTT)	GDM status Fasting glucose HOMA-IR	NR	hPL highest in GDM women pregnant with female fetus, then control with female fetus, then GDM with male fetus, then control with male fetus (all sig). hPL levels NS related to fasting glucose in all 4 groups. hPL levels inversely related to HOMA-IR in all 4 groups, but sig only in non-GDM women with female fetus (r = −0.790, *p* = 0.001).	hPL highest in GDM women carrying female infants (note groups not matched for BMI or other key baseline characteristics). No clear overall relationships between hPL and fasting glucose. Suggestion of inverse relationship between hPL and IR but sig only in one subgroup.	High
Catalano et al., 1993 [37] USA	Cross-sectional	*n* = 38 women with abnormal screening GCT	OGTT performed twice, 1 week apart, at 27–30 weeks; hPL sampled alongside	GDM status	NDDG, but deemed abnormal if only one value exceeded thresholds	Study focused on how gestational hormones may influence OGTT reproducibility. hPL NS diff at either first or second OGTT in either women with ‘definite’ non-GDM (2 normal results), ‘definite’ GDM (2 abnormal results) or those with discordant results (one normal and one abnormal result).	hPL does not appear to be a hormonal factor influencing OGTT reproducibility.	Low
Catalano et al., 2002 [26] USA	Longitudinal observational	*n* = 5 obese GDM *n* = 4 obese controls	hPL sampled at: 12–14 weeks 34–36 weeks	GDM status	Carpenter-Coustan	hPL NS diff between GDM women and controls: in both early preg (mean ±SEM of GDM vs. controls 0.85 ± 0.36 vs. 0.89 ± 0.33 µg/mL) and late preg (GDM vs. controls 7.1 ± 1.13 vs. 8.00 ± 1.19 µg/mL), *p* = 0.3 for both.	hPL NS diff between GDM women and control women in either early or late preg.	High
De Hertogh et al., 1976 [16] Belgium	Longitudinal observational	*n* = 19 GDM *n* = 22 controls	hPL sampled at: 5–8 weeks 9–12 weeks 13–16 weeks 17–20 weeks 21–24 weeks 25–28 weeks 29–32 weeks 33–36 weeks 37–40 weeks	GDM status	100 g OGTT. 0, 30, 60, 120, 180 min; thresholds 5.0/8.9/8.3/6.7/5.5 mmol/L. ≥2 high for dx	Mean hPL sig lower in GDM than control group at 17–20 weeks only. At all other timepoints, hPL NS diff between GDM and controls.	hPL not consistently sig diff between GDM and control women in serial preg sampling.	High
Grigorakis et al., 2000 [31] Greece	Cross-sectional	*n* = 15 GDM *n* = 26 controls	One-off hPL sample at 28–32 weeks at time of OGTT	GDM status	ADA	Mean hPL NS diff between GDM and controls (GDM 4.9 ± 1.3 vs. controls 4.3 ± 1.4 µg/mL).	hPL NS diff between GDM and controls in late preg. Any contribution of hPL to GDM pathophysiology ‘likely to be weak’.	Moderate
Henderson et al., 1998 [34] USA	Cross-sectional	*n* = 257 women, of whom *n* = 57 had abnormal screening GCT; and *n* = 25 then had abnormal OGTT (i.e., GDM)	One-off hPL sample at time of GCT (exact timepoint NR but presume early 3rd trimester)	GDM status	Carpenter-Coustan	Of women who had an abnormal GCT, hPL sig higher in women who proceeded to abnormal OGTT than in those who had a subsequent normal OGTT; 5.85 ± 2.55 µg/mL vs. 3.38 ± 1.40, *p* = 0.034. NS diff in hPL between women with normal GCT (4.68 ± 1.64 µg/mL), and those who had abnormal GCT but ultimately went on to have normal OGTT. *n* = 11 women had normal GCT but subsequently delivered an infant > 4 kg, in them mean hPL at time of GCT had been similar to the GDM group (5.83 ± 1.29 µg/mL).	hPL in appeared a helpful adjunct to GCT, and seemed to help with predicting those who would go on to have positive OGTT. hPL in normoglycaemic women who proceeded to deliver macrosomic infants had retrospectively been similar to that of GDM women–potential for hPL as a risk predictor for macrosomia, or of GDM ‘missed’ by OGTT?	Moderate
Kirwan et al., 2002 [39] USA	Longitudinal observational	*n* = 5 obese GDM *n* = 5 lean controls *n* = 5 obese controls	hPL sampled at: pre-conception 10–12 weeks 34–36 weeks	Insulin sensitivity	Carpenter-Coustan	Insulin sensitivity measured via clamp in early and late preg. Late preg insulin sensitivity found to be NS related to hPL levels (r = −0.24, *p* = 0.39).	hPL levels in late preg did not appear to be sig related to the degree of preg-induced insulin resistance in GDM or non-GDM women (main sig findings of the trial related to TNF-α, which did emerge as sig related to insulin resistance).	Moderate
Kuhl et al., 1975 [36] Denmark	Cross-sectional	*n* = 11 GDM *n* = 9 controls	One-off hPL sample at 34–35 weeks at time of OGTT	GDM status OGTT glucose	At least 2 values on OGTT that were >3 SD above mean of authors’ previous normal preg population	Mean fasting hPL NS diff in GDM vs. controls (6.2 ± 2.3 vs. 6.7 ± 1.3 µg/mL). Mean hPL at 3 h of OGTT NS diff in GDM vs. controls (6.1 ± 2.6 vs. 6.2 ± 1.1 µg/mL). No sig alteration in hPL over course of OGTT in either group. No difference in shape of hPL curves alongside OGTT in GDM vs. controls.	hPL NS diff between GDM and controls. Previous literature suggestive of high late preg hPL values in T1DM patients may not apply to mild GDM. hPL does not appear to be sig altered by minor physiological fluctuations in plasma glucose, such as with OGTT.	Moderate
Lopez-Espinoza et al., 1986 [18] Scotland	Longitudinal observational	*n* = 8 early GDM on insulin *n* = 14 controls	For GDM, hPL sampled fortnightly 12–32 wk, then weekly until delivery. For controls, hPL sampled monthly	GDM status Plasma glucose Insulin requirements	WHO 1980	Mean hPL higher in GDM than controls in 2nd trimester (mean ±SEM 2.3 ± 0.4 vs. 1.3 ± 0.1 µg/mL, *p* < 0.05). In early and late third trimester, hPL also showed a trend to being higher in GDM than controls (early 3rd trimester 6.3 ± 0.7 vs. 5.4 ± 0.5; late 7.7 ± 0.9 vs. 6.5 ± 0.6) but did not achieve sig. hPL not related to plasma glucose or insulin requirements in GDM.	Suggestion of higher hPL levels in GDM than controls across gestation in this study, although sig only in second trimester. No apparent relationship between hPL and either plasma glucose or insulin requirements in GDM.	Moderate
Luthman et al., 1994 [38] Sweden	Cross-sectional	*n* = 12 GDM *n* = 12 controls	hPL sampling across standard breakfast (0 to 120 min), at 29–38 weeks	GDM status Plasma glucose	WHO 1980	hPL NS diff between GDM and control women at all timepoints. hPL levels NS altered by glucose excursions after standard meal (in either group).	hPL unaltered by meal ingestion, and no different between GDM and control women, in third trimester.	Moderate
Ngala et al., 2017 [29] † Ghana	Longitudinal observational	*n* = 200 preg women in 1st trimester: *n* = 50 ‘low risk’ for diabetes and *n* = 150 ‘standard risk’. *n* = 12 later developed GDM	One-off hPL sample at 24–28 weeks	GDM status GDM risk Fasting glucose, insulin, IR, BMI, total cholesterol, Tg	ADA	When *n* = 12 GDM compared to all *n* = 138 non-GDM, NS diff between hPL levels at 24–28 weeks (*p* = 0.155). When *n* = 12 GDM compared to subgroup of *n* = 50 women deemed ‘low risk of diabetes’, hPL sig lower in the GDM women (*p* < 0.0001). In multiple logistic regression, hPL at 24–28 weeks did not emerge as a sig predictor of GDM risk. All metabolic parameters NS related to 24–28 week hPL in either GDM or non-GDM women.	hPL NS different between GDM and non-GDM women at 24–28 weeks, and did not emerge as a sig predictor of GDM risk. hPL at 24–28 weeks not linked to other metabolic parameters including BMI, lipids, fasting glucose or insulin.	High
Persson et al., 1975 [22] Sweden	Cross-sectional	*n* = 8 GDM *n* = 5 controls	Five measurements of hPL over one 8 h period at 34–37 weeks	GDM status Blood glucose FFAs, glycerol Ketones Insulin	IVGTT in third trimester in at-risk women, own criteria	Mean hPL over the 8 h sampling period was NS diff between GDM vs. control women. hPL changes over the sampling period bore no apparent relationship to changes in glucose, FFAs, ketones, or insulin over the sampling period.	hPL in over 8 h in third trimester NS diff between GDM and controls. hPL not clearly related to insulin or glucose dynamics over an 8 h period in GDM or controls.	High
Rasanen et al., 2013 [28] Finland	Case-control	*n* = 90 GDM *n* = 92 controls	GDM cases and controls identified in late preg. hPL at 5–13 weeks then compared between cases and controls	GDM status GDM risk	ADA, but deemed abnormal if only one value exceeded thresholds	Median hPL in first trimester sig higher in women who would go on to get GDM than in controls (GDM vs. controls 0.34 vs. 0.22 µg/mL, *p* < 0.001). AUC on ROC curve for GDM prediction with threshold 0.80 ng/mL: sensitivity 28%, specificity 90%, AUC 0.63 (0.55–0.71, *p* < 0.001), i.e., only very marginal classification benefit.	First trimester hPL was sig diff between women who would go on to get GDM and those who would not (i.e., sig association with GDM risk, higher hPL in GDM than controls). However, degree of separation between distributions of hPL in cases and controls was not adequate for use as screening test (low AUC; poor classification performance).	Low
Retnakaran et al., 2016 [32] Canada	Cross-sectional	*n* = 105 GDM *n* = 290 controls	One-off hPL sample at 29–30 weeks, at time of OGTT	GDM status AUC glucose, Matsuda index, HOMA-IR, fasting insulin, ISSI-2, and IGI/HOMA-IR	NDDG	Median hPL NS diff between GDM women and controls (2.0 vs. 1.9µg/mL, *p* = 0.1). No variable showed a sig association with hPL in either GDM or control women, before or after adjustment for key covariates.	hPL NS diff between GDM and non-GDM women at time of OGTT. hPL NS associated with AUC glucose in either GDM or non-GDM women. hPL NS associated with other markers of insulin sensitivity or beta cell function in either GDM or non-GDM women. Data suggests that circulating hPL concentrations may not provide direct insights on maternal glucose homeostasis.	Moderate
Samaan et al., 1985 [35] USA	Cross-sectional	*n* = 14 GDM *n* = 17 controls	One-off hPL sample at delivery	GDM status	NR	Mean hPL at time of delivery sig higher in GDM than controls (8.85 ± 1.4 vs. 6.8 ± 2.1 µg/mL, *p* < 0.001).	hPL at time of delivery sig higher in diet-controlled GDM women than in controls.	High
Surmaczynska et al., 1974 [33] USA	Cross-sectional	*n* = 13 GDM *n* = 33 controls	One off hPL sample at 30–40 weeks, at time of OGTT	GDM status OGTT	O’Sullivan and Mahan	Mean baseline hPL NS diff between GDM and controls (mean ±SEM 8.8 ± 0.64 vs. 8.4 ± 0.45 µg/mL). Very marginal hPL decrement with 100 g OGTT seen in overall cohort; magnitude NS diff between GDM and controls.	hPL at 30–40 weeks NS diff between GDM and non-GDM women. hPL dipped slightly with OGTT in cohort overall, magnitude no diff between GDM and non-GDM women.	Moderate

Abbreviations: hPL = human placental lactogen, NS = non-significant, sig = significant, NR = not reported, GDM = gestational diabetes mellitus, SEM = standard error of the mean, IUGR = intra-uterine growth restriction, PIH = pregnancy-induced hypertension, NDDG = National Diabetes Data Group, NGT = normal glucose tolerance, ADA = American Diabetes Association, GCT = glucose challenge test, OGTT = oral glucose tolerance test, IVGTT = intravenous glucose tolerance test, WHO = World Health Organisation, T1DM = type 1 diabetes mellitus, BMI = body mass index, IR = insulin resistance, Tg = triglycerides, AUC = area under the curve, ROC = receiver operating characteristic, HOMA-IR = Homeostatic Model Assessment for Insulin Resistance, ISSI = insulin-secretion sensitivity index, IGI = insulinogenic index, TNFα = tumor necrosis factor-alpha, USA = United States of America. Data are presented as mean ± SD unless otherwise specified in the table. † **=** The 2017 study by Ngala et al. [29] met inclusion criteria for the review, but serious methodological concerns were raised about this paper during the review process. In particular, the authors’ tabulated hPL values for 24–28 weeks (in µg/mL) are approximately ten times greater than the expected physiological ranges at this gestation; and it is suspected that an error has occurred with assay methodology or unit conversion. The authors were contacted for comment, but no reply had been received at the time of manuscript submission. Results of this study need to be interpreted with extreme caution, and the study was omitted from meta-analysis on this basis. Significant methodological concerns were also raised about the 2021 paper by Al-Hussein et al. [30], in which hPL values were presented without any units and with no indication of gestational age at time of sampling. Again, authors did not reply to an invitation to clarify and the study was excluded from meta-analysis.

**Table A3:** Studies examining hPL in relation to glycaemic or insulin-related parameters in pregnancy/postpartum—5 studies.

Author and Year; Country of Origin	Design	Participants and Sample Size	Methodology and hPL Pregnancy timepoints	Metabolic Parameters Analysed	Results	Authors’ Conclusions	Risk of Bias Rating
Benny et al., 1980 [40] UK	Cross-sectional	*n* = 21 women with normal OGTT in preg, none obese (*n* = 10 Hindi vegetarians, *n* = 11 Caucasian omnivores)	Eleven serial measures of hPL over one 24 h period at 36–39 weeks	Insulin Glucose	Insulin and glucose sampled serially across 24 h period, as was hPL. hPL peak (at 0500h after overnight fast) coincided with nadir of insulin and glucose. No direct correlation of individuals’ hPL levels with insulin or glucose. However, Hindi women were found to have sig higher mean glucose than Caucasian women. This was unlikely to be mediated by hPL because hPL was sig lower in Hindi than Caucasian women (5.79 ± 0.05 vs. 6.11 ± 0.05 µg/mL; *p* < 0.01). Lower hPL in the Hindi women likely related to sig lower placental masses.	hPL appeared to peak after overnight fast in pregnancy, temporally coinciding with time of lowest glucose and insulin. Hindi women had higher mean glucose levels in third trimester than Caucasians, but lower hPL levels (maybe related to smaller placentas)–so hPL unlikely to be driving glycaemic differences.	Moderate
Enzi et al., 1980 [41] Italy	Longitudinal observational	*n* = 50 healthy preg women	One-off hPL sample at 34–35 weeks	AUC glucose AUC insulin	hPL at 34/40 positively related to maternal AUC glucose at 34/40, r = 0.62, *p* < 0.001. hPL at 34/40 positively related to maternal AUC insulin at 34/40, r = 0.31, *p* < 0.05	hPL at 34 weeks positively related to maternal AUC insulin and AUC glucose, suggesting diabetogenic effects.	Low
Fairweather et al., 1971 [42] UK	Longitudinal observational	*n* = 33 healthy preg women	hPL sampling: 6–12 weeks 13–19 weeks 20–25 weeks 26–30 weeks 31–32 weeks 33–34 weeks 35–36 weeks 37–38 weeks 39–40 weeks 41–42 weeks	Glucose NEFAs	NS direct relationship between glucose and hPL levels at a given time in a given patient. Positive relationship between hPL and NEFA levels, r = 0.24, *p* < 0.01; i.e., higher hPL levels at at a given time in a given patient tended to be assoc with higher NEFA levels.	hPL showed NS relationship to glucose levels within a patient at any given time, but higher hPL levels tended to be associated with higher levels of NEFAs. hPL appears to have anti-insulin, diabetogenic effects that promote mobilisation of FFAs and reduce maternal glucose utilisation, sparing glucose to meet fetal demands.	Moderate
Retnakaran et al., 2016 [44] Canada	Longitudinal observational	*n* = 301 NGT *n* = 60 pre-diabetes *n* = 6 DM (based on OGTT at 3 months postpartum)	hPL sampled at time of OGTT in late second trimester of preg, but then analysed in relation to postpartum metabolic status	Maternal diabetes category at 3 mo postpartum Glycaemic markers at 3 mo postpartum Risk of pre-DM or DM at 3 mo postpartum	hPL in late preg had been no diff between who went on to be NGT at 3 mo postpartum, those with pre-diabetes at 3 mo postpartum, and those with DM at 3 mo postpartum (median hPL in µg/mL = NGT 2.0 vs. pre-DM 2.0 vs. DM 1.5, *p* = 0.312). On multivariate regression, hPL in late preg not independently related to any glycaemic markers (log Matsuda index, log HOMA-IR, log ISSI-2, log IGI/HOMA-IR, fasting glucose, AUC glucose) at 3 mo post partum. On multivariate regression, hPL in late preg not an independent predictor of the risk of persistent dysglycaemia at 3 mo postpartum.	hPL in late preg had been no diff between those with normal glucose tolerance at 3 mo postpartum and those with postpartum pre-DM or DM. hPL in late preg was not an independent determinant of insulin resistance or beta-cell function at 3 mo postpartum. hPL in late preg was not an independent predictor of the risk of pre-DM or DM at 3 mo postpartum.	Moderate
Scott et al., 1992 [43] UK	Cross-sectional	*n* = 127 healthy preg women (*n* = 97 European, *n* = 30 Asian)	One-off hPL sample at 29 weeks, at time of OGTT	2 h insulin on OGTT 2 h glucose on OGTT	No relationship to hPL in either ethnic group. No relationship to hPL in either ethnic group.	hPL not related to either 2 h OGTT insulin or 2 h OGTT glucose in either race in this study. hPL does not clearly play a role in modifying insulin action.	Low

Abbreviations: hPL = human placental lactogen, NS = non-significant, sig = significant, AUC = area under the curve, NEFAs = non-esterified fatty acids, FFAs = free fatty acids, NGT = normal glucose tolerance, OGTT = oral glucose tolerance test, DM = diabetes mellitus, HOMA-IR = Homeostatic Model Assessment for Insulin Resistance, ISSI = insulin-secretion sensitivity index, IGI = insulinogenic index, UK = United Kingdom. Data are presented as mean ± SD unless otherwise specified in the table.

**Table A4:** Studies examining hPL in relation to body mass index and/or gestational weight gain in pregnancy—4 studies.

Author and Year; Country of Origin	Design	Participants and Sample Size	Methodology	Metabolic Parameters Analysed	Results	Authors’ Conclusions	Risk of Bias Rating
Al-Hussein et al., 2021 [30] † Iraq	Cross-sectional	*n* = 40 GDM (20 male fetus, 20 female) *n* = 40 controls (20 male fetus, 20 female)	One-off hPL sampling, presumably > 24–28 weeks after OGTT	Maternal BMI	Maternal BMI NS rel to maternal hPL level in any group.	No sig relationship between maternal BMI and hPL demonstrated in any study subgroup.	High
Enzi et al., 1980 [41] Italy	Longitudinal observational	*n* = 50 healthy preg women	One-off hPL sample at 34–35 weeks	Maternal GWG	Maternal hPL at 34 weeks NS diff between mothers in excessive GWG group (gained >20% IBW, mean 16.5 ± 1.4 kg, *n* = 23) and those in normal GWG group (gained <20% IBW, mean 8.7 ± 0.5 kg, *n* = 27). Excessive GWG mean hPL 7.7 ± 1.5 µg/mL vs. normal GWG 6.3 ± 1.1 µg/mL.	hPL at 34 weeks NS diff between mothers who had normal GWG and those with excessive GWG.	Low
Lin et al., 1976 [45] USA	Cross-sectional	*n* = 187 healthy preg women	One-off hPL sample near term (within one week of delivery)	Maternal weight	Maternal weight sig inversely related to maternal hPL at term (r = −0.28, *p* <0.01).	Maternal weight at term sig inversely related to hPL concentration at term. Authors suggest this might be dilutional effect (?more tissue space in larger women).	Low
McCarrick et al., 1979 [46] USA	Longitudinal observational	*n* = 290 preg women with preg risk factors (eg prev or current GDM, PET, previous losses, IUGR)	Serial hPL sampling across third trimester 33–40 weeks (approx. 4–5 per woman)	Weight category	Obese women (>72.6 kg at 16 weeks) were over-represented in “group 3” of the study, *n* = 44, all of whom had normal estrogen but low hPL (50% women in that group obese vs. 25% obese in other groups, *p* < 0.001). These women were at an increased risk of preg complications such as fetal death or SGA (34.1% had complications) compared to those with normal hPL and estrogen (group 1, of whom 4.7% had complications) although not as high as those with both low hPL and low estrogen (group 2, of whom 71.4% had complications.)	Obese women sig proportionally over-represented in the group of women with low hPL but normal estrogen in the study. Authors conclusion was that obesity may impact on hPL regulation and activity. Note that direct impact of the complications themselves on hPL levels was not considered.	High

Abbreviations: hPL = human placental lactogen, GDM = gestational diabetes mellitus, sig = significant, NS = non-significant, IBW = ideal body weight, BMI = body mass index, GWG = gestational weight gain, PET = pre-eclampsia toxaemia, NS = non-significant, sig = significant, SGA = small for gestational age, IUGR = intra-uterine growth restriction, USA = United States of America. Data are presented as mean ± SD unless otherwise specified in the table. † = Note methodological concerns, see Table A2 footnote.

**Table A5:** Studies examining hPL in relation to fetal, neonatal or placental outcomes in pregnancies affected by maternal pre-gestational/gestational diabetes—7 studies.

Author and Year; Country of Origin	Design	Participants and Sample Size	Methodology	Fetal or Placental Outcomes	Results	Authors’ Conclusions	Risk of Bias Rating
Botta et al., 1984 [14] Italy	Longitudinal observational	*n* = 15 T1DM *n* = 10 controls	Serial hPL sampling across preg	Placental weight Birthweight	Placental weight positively correlated to week 36 hPL (r = 0.368) across whole cohort, although not sig (*p*-value NR). Birthweight positively correlated to week 36 hPL (r = 0.319) across whole cohort, although NS (*p*-value NR).	Late pregnancy hPL positively related to both placental mass and birthweight across combined cohort, although short of sig.	Moderate
Lopez-Espinoza et al., 1986 [18] Scotland	Longitudinal observational	*n* = 15 T1DM *n* = 8 GDM *n* = 14 controls	Serial hPL sampling across preg	Placental weight	Positively related to pre-delivery (>37 week) hPL in T1DM (r = 0.8, *p* < 0.01). Positively related to third trimester (<37 week) hPL in GDM (r = 0.6, *p* < 0.05). Positively related to pre-delivery (>37 week) hPL in controls (r = 0.6, *p* < 0.05).	Late preg hPL levels strongly positively correlated with placental weight in control, T1DM and GDM women.	Moderate
Luthman et al., 1994 [38] Sweden	Cross-sectional	*n* = 12 GDM *n* = 12 controls	One-off hPL sample at 29–38 weeks	Birthweight	Positively correlated to third trimester hPL in GDM cohort (r = 0.59, *p* < 0.05). No such relationship found in controls or in overall cohort.	Late pregnancy hPL positively correlated with birthweight in the GDM cohort.	Moderate
Pedersen et al., 1986 [48] Denmark	Cross-sectional	*n* = 26 T1DM	One-off hPL sample at 7–16 weeks	Early fetal growth	√hPL in early pregnancy related to menstrual age corrected by 90% of the growth delay (growth delay = diff in days between menstrual age and USS CRL age). √hPL (mg/l) = −0.541 + 0.0142 menstrual age (days) −0.0128 delay (days).	Authors had previously noted that size of T1DM pregnancies (by CRL on USS) may lag by several days behind the age calculated from LMP. Here, hPL could be best mathematically related to menstrual age when it was corrected by this delay. Given hPL reflects functional placental mass, this suggests that the observed growth delay in early T1DM pregnancies is accompanied by a delay in placental development.	High
Samaan et al., 1985 [35] USA	Cross-sectional	*n* = 14 GDM *n* = 17 controls	One-off hPL sample at time of delivery	Birthweight	Across whole cohort (GDM women, controls; and a third group of women with preterm birth, *n* = 15); NS correlation between maternal hPL and neonatal weight. Not described for GDM cohort individually.	NS relationship between hPL at time of delivery and birthweight across combined cohort of GDM, preterm birth and control women.	High
Small et al., 1987 [47] Scotland	Longitudinal observational	*n* = 20 T1DM with macrosomia (birthweight >90% for gestation) *n* = 20 matched T1DM without macrosomia	One-off hPL sample at 34 weeks	Birthweight class	T1DM group with macrosomia (mean birthweight 3.96 kg at 37 weeks) had sig higher hPL at 34 weeks than matched T1DM preg without macrosomia (mean birthweight 3.05 kg at 37 weeks). Macrosomia group mean hPL = 8.3 ± 2.3 µg/mL vs. non-macrosomia group mean hPL = 6.5 ± 2.3 µg/mL; *p* < 0.005.	hPL at 34 weeks was sig higher in *n* = 20 T1DM women who gave birth to macrosomic infants than in *n* = 20 T1DM who gave birth to normal weight infants. Authors suggested that hPL may help with detection of macrosomia early in the third trimester.	Low
Stewart et al., 1989 [20] UK	Longitudinal observational	*n* = 40 T1DM *n* = 69 controls	Serial hPL sampling across preg	Placental weight Birthweight	hPL at 32 or 36 weeks NS related to placental weight in T1DM group as a whole, using sig value of *p* < 0.01. hPL at 32 or 36 weeks NS related to birthweight in T1DM group as a whole, using sig value of *p* < 0.01. However, was a positive correlation between birthweight and 32 week hPL if *p* < 0.02 accepted (r = 0.48, *p* < 0.02). Both birth and placental weight corrected for maternal parity, maternal stature, infant sex and length of gestation.	NS relationship seen between hPL and placental mass, or hPL and birthweight, in T1DM in this cohort (using stringent sig threshold of *p* < 0.01). Did see a positive relationship between hPL and birthweight in T1DM group when sig level of *p* < 0.02 accepted.	Low

Abbreviations: hPL = human placental lactogen, GDM = gestational diabetes mellitus, sig = significant, NS = non-significant, NR = not reported, GA = gestational age, T1DM = type 1 diabetes mellitus, USS = ultrasound scan, CRL = crown-rump length, LMP = last menstrual period, USA = United States of America, UK = United Kingdom. Data are presented as mean ± SD unless otherwise specified in the table.
