# Supplementary material for: Human Placental Lactogen in Relation to Maternal Metabolic Health and Fetal Outcomes: A Systematic Review and Meta-Analysis
Source: Int J Mol Sci. 2022 Dec 9;23(24):15621. doi: 10.3390/ijms232415621 (PMC9779646; doi:10.3390/ijms232415621)

Supplementary material 2: Funnel plots

Funnel plot for meta-analysis of hPL levels in T1DM vs non-diabetic control women in late pregnancy (>24 weeks) – 4 studies

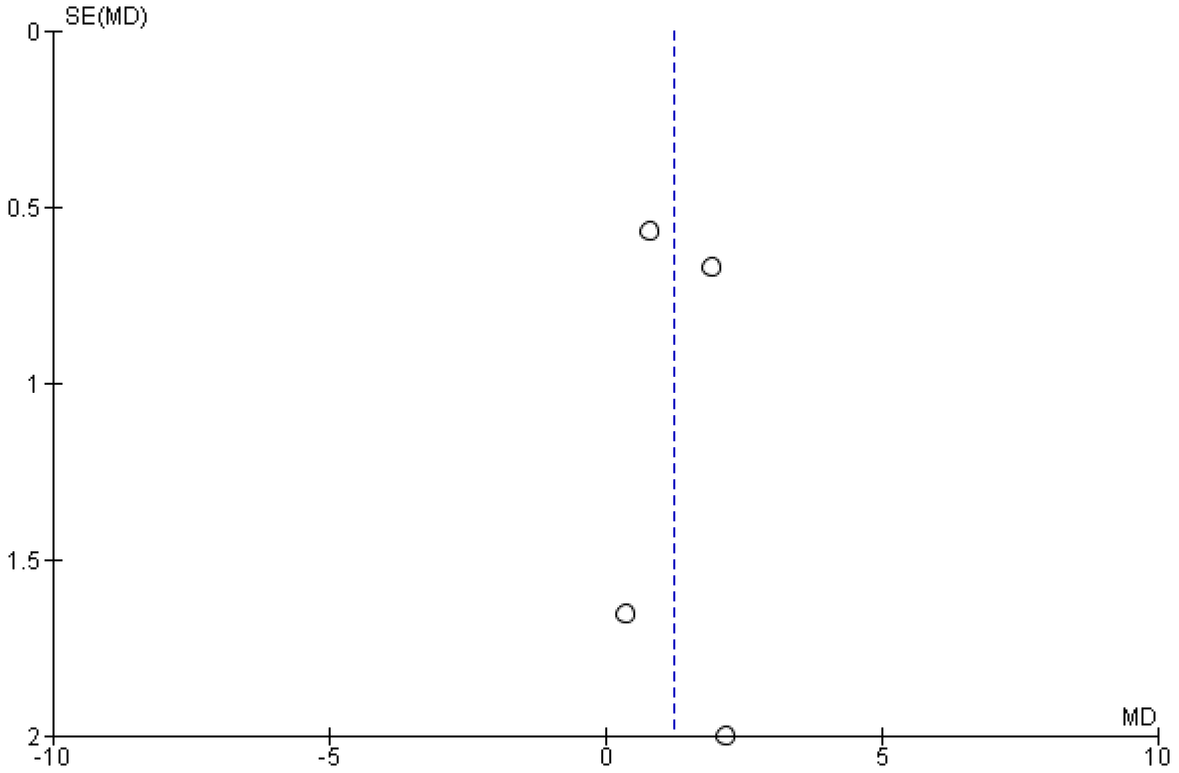

Funnel plot for meta-analysis of hPL levels in GDM vs non-diabetic control women in early pregnancy (≤24 weeks) – 3 studies

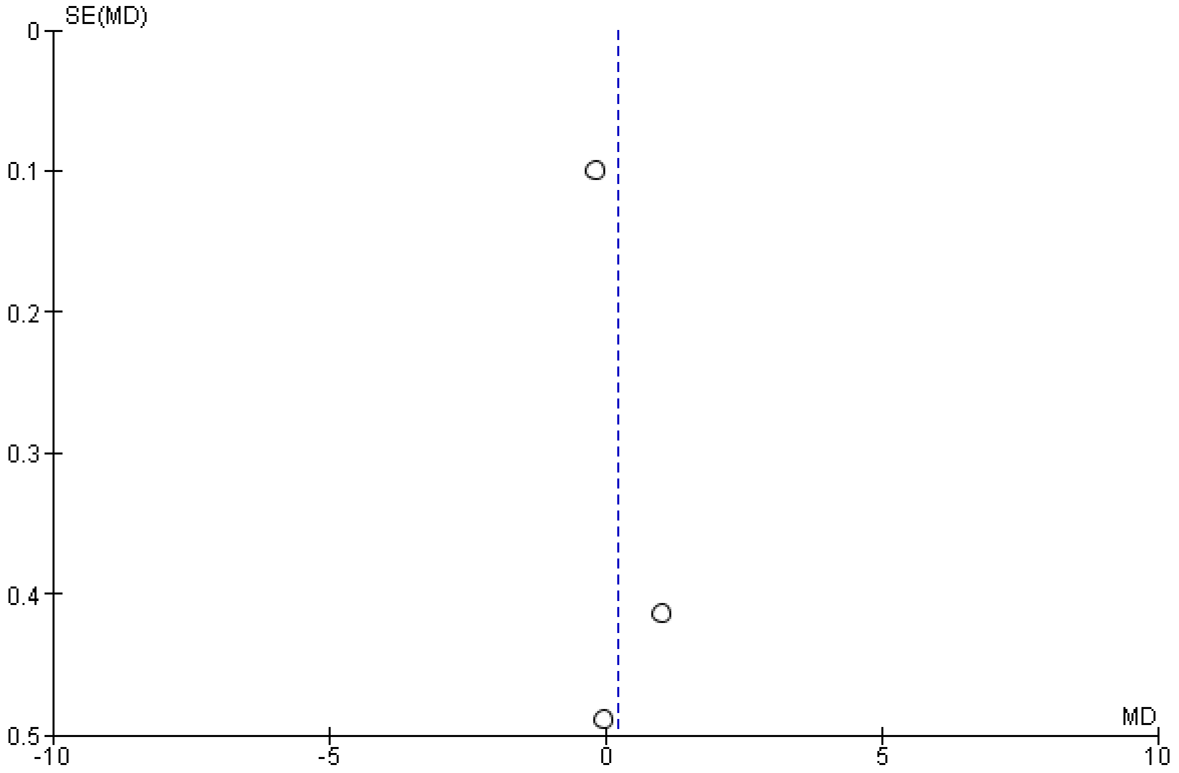

Funnel plot for meta-analysis of hPL levels in GDM vs non-diabetic control women in late pregnancy (>24 weeks) – 10 studies

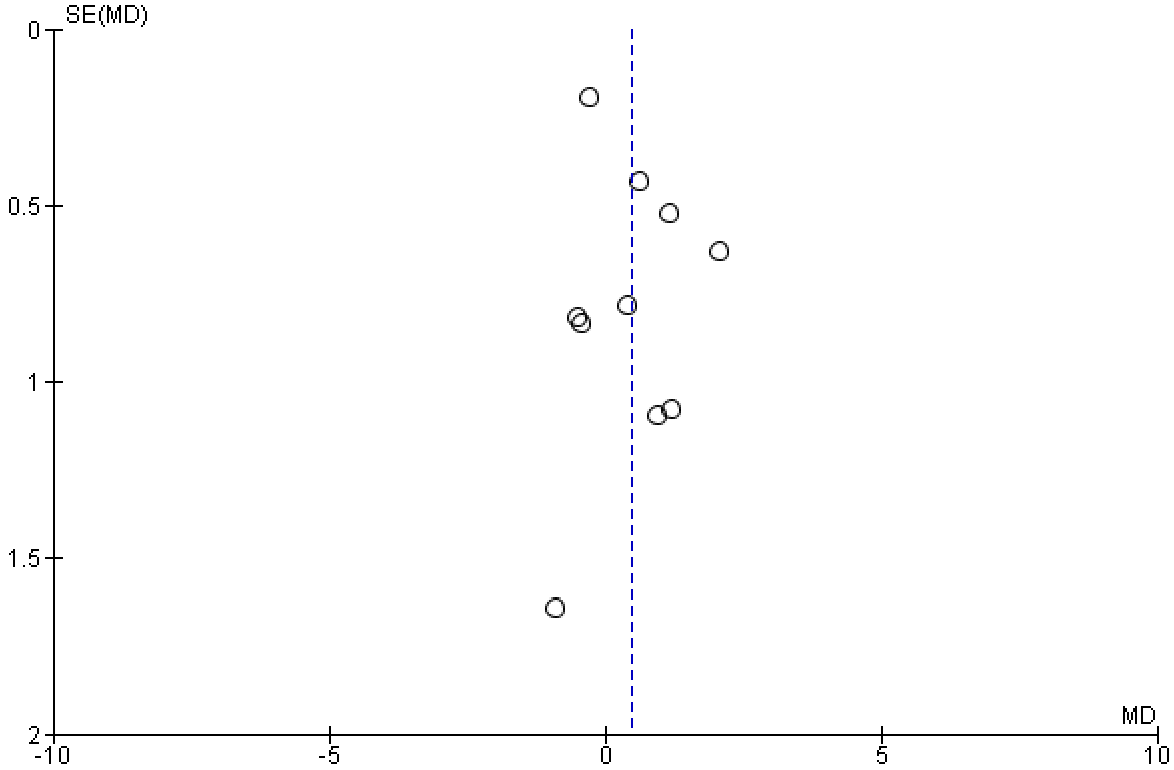

Supplement: Supplementary file 1 [file ijms-23-15621-s001.zip › Supplementary material for hPL paper 2.pdf]
